# Structural and Dynamical Order of a Disordered Protein: Molecular Insights into Conformational Switching of PAGE4 at the Systems Level

**DOI:** 10.3390/biom9020077

**Published:** 2019-02-22

**Authors:** Xingcheng Lin, Prakash Kulkarni, Federico Bocci, Nicholas P. Schafer, Susmita Roy, Min-Yeh Tsai, Yanan He, Yihong Chen, Krithika Rajagopalan, Steven M. Mooney, Yu Zeng, Keith Weninger, Alex Grishaev, José N. Onuchic, Herbert Levine, Peter G. Wolynes, Ravi Salgia, Govindan Rangarajan, Vladimir Uversky, John Orban, Mohit Kumar Jolly

**Affiliations:** 1 Center for Theoretical Biological Physics, Rice University, Houston, TX 77030, USA; xclin@mit.edu (X.L.); fb20@rice.edu (F.B.); npschafer@gmail.com (N.P.S.); susmitajanaroy@gmail.com (S.R.); mytsai886@gmail.com (M.-Y.T.); herbert.levine@rice.edu (H.L.); pwolynes@rice.edu (P.G.W.); 2 Department of Physics and Astronomy, Rice University, Houston, TX 77005, USA; 3 Department of Chemistry, Massachusetts Institute of Technology, Cambridge, MA 02139, USA; 4 Department of Medical Oncology and Therapeutics Research, City of Hope National Medical Center, Duarte, CA 91010, USA; rsalgia@coh.org; 5 Department of Chemistry, Rice University, Houston, TX 77005, USA; 6 Department of Chemistry, Tamkang University, New Taipei City 25137, Taiwan; 7 Institute for Bioscience and Biotechnology Research, University of Maryland, Rockville, MD 20850, USA; hey@umd.edu (Y.H.); yihong@umd.edu (Y.C.); agrishaev@ibbr.umd.edu (A.G.); 8 Division of Biological Sciences, University of California, San Diego, La Jolla, CA 92093, USA; krrajagopalan@ucsd.edu; 9 Department of Biology, University of Waterloo, Waterloo, ON N2L 3G1, Canada; steve.mooney@uwaterloo.ca; 10 Department of Urology, The First Hospital of China Medical University Shenyang, Shenyang 110001, China; zengyud@hotmail.com; 11 Department of Physics, North Carolina State University, Raleigh, NC 27695, USA; krwening@ncsu.edu; 12 National Institute of Standards and Technology, Gaithersburg, MD 20899, USA; 13 Department of BioSciences, Rice University, Houston, TX 77005, USA; 14 Department of Mathematics, Indian Institute of Science, Bangalore 560012, India; rangaraj@iisc.ac.in; 15 Center for Neuroscience, Indian Institute of Science, Bangalore 560012, India; 16 Department of Molecular Medicine, Morsani College of Medicine, University of South Florida, Tampa, FL 33612, USA; vuversky@health.usf.edu; 17 Laboratory of New methods in Biology, Institute for Biological Instrumentation, Russian Academy of Sciences, 142290 Pushchino, Moscow Region, Russia; 18 Department of Chemistry and Biochemistry, University of Maryland, College Park, MD 20742, USA; 19 Center for BioSystems Science and Engineering, Indian Institute of Science, Bangalore 560012, India; 20 Department of Physics, Northeastern University, Boston, MA 02115, USA

**Keywords:** PAGE4, intrinsically disordered proteins, conformational plasticity, order–disorder transition, phosphorylation

## Abstract

Folded proteins show a high degree of structural order and undergo (fairly constrained) collective motions related to their functions. On the other hand, intrinsically disordered proteins (IDPs), while lacking a well-defined three-dimensional structure, do exhibit some structural and dynamical ordering, but are less constrained in their motions than folded proteins. The larger structural plasticity of IDPs emphasizes the importance of entropically driven motions. Many IDPs undergo function-related disorder-to-order transitions driven by their interaction with specific binding partners. As experimental techniques become more sensitive and become better integrated with computational simulations, we are beginning to see how the modest structural ordering and large amplitude collective motions of IDPs endow them with an ability to mediate multiple interactions with different partners in the cell. To illustrate these points, here, we use Prostate-associated gene 4 (PAGE4), an IDP implicated in prostate cancer (PCa) as an example. We first review our previous efforts using molecular dynamics simulations based on atomistic AWSEM to study the conformational dynamics of PAGE4 and how its motions change in its different physiologically relevant phosphorylated forms. Our simulations quantitatively reproduced experimental observations and revealed how structural and dynamical ordering are encoded in the sequence of PAGE4 and can be modulated by different extents of phosphorylation by the kinases HIPK1 and CLK2. This ordering is reflected in changing populations of certain secondary structural elements as well as in the regularity of its collective motions. These ordered features are directly correlated with the functional interactions of WT-PAGE4, HIPK1-PAGE4 and CLK2-PAGE4 with the AP-1 signaling axis. These interactions give rise to repeated transitions between (high HIPK1-PAGE4, low CLK2-PAGE4) and (low HIPK1-PAGE4, high CLK2-PAGE4) cell phenotypes, which possess differing sensitivities to the standard PCa therapies, such as androgen deprivation therapy (ADT). We argue that, although the structural plasticity of an IDP is important in promoting promiscuous interactions, the modulation of the structural ordering is important for sculpting its interactions so as to rewire with agility biomolecular interaction networks with significant functional consequences.

## 1. Introduction

Strictly speaking, both canonically folded proteins and intrinsically disordered proteins (IDPs) lack unique three-dimensional (3D) structures [1]. Folded proteins undergo structural fluctuations on timescales ranging from femtoseconds to seconds, while IDPs exhibit dynamics on times ranging up to milliseconds. Thermal motions explore low-lying states on the energy landscapes of proteins. These low-lying states are simply more numerous and more structurally diverse in the case of IDPs. Despite their lack of stable structures, IDPs are indispensable in regulating various cellular functions. The greater structural plasticity of IDPs facilitates their interactions with multiple binding partners, leading to “one-to-many” and “many-to-one” binding strategies [2]. This functional plasticity coming from their structural diversity allows IDPs to function as hubs in protein–protein interaction networks, thereby regulating in a more complex fashion cellular decision making [3,4].

Although some studies have shown that IDPs can remain structurally disordered even when they interact with a cognate partner [5], this disorder is far from the complete randomness envisioned in elementary polymer physics models as self-avoiding random walks in 3D space, without any structural preferences [6]. In contrast to that elementary picture, many IDPs exhibit both local structural and dynamical ordering. IDPs undergo transitions among a diversity of metastable states that may be differentially stabilized by binding to interaction partners [7]. By being shrouded by a relatively high degree of structural plasticity, this ordering is difficult to discern using standard biophysical techniques. Furthermore, due to their intrinsically diverse conformational ensembles, IDPs are more susceptible to perturbation when compared to folded proteins. They therefore undergo larger structural modulation by the binding of ligands or by post-translational modifications (PTMs) [8,9], which can thereby perturb the balance between different functional states. The plasticity of IDPs is reflected by a set of activities of proteins that are driven by entropy [10,11,12]. Furthermore, it has recently been recognized that some IDPs can engage in polyvalent and highly dynamic interactions that drive large-scale liquid–liquid phase transitions that may be crucial in the generation of membrane-less organelles [13,14,15,16].

The existence of plasticity in structural conformations of proteins has long been recognized. It is now well-established that many proteins are polymorphic, changing from one folded state to another as a part of their functional repertoire [17]. Conformational switching, as well as the associated allosteric transitions, enables different functional behaviors that depend on environmental conditions. This conformational plasticity is especially prominent in metamorphic proteins [18], where multiple conformations with widely different structures exist for a single encoded sequence [19,20,21]. Therefore, IDPs do not stand entirely apart from their folded brethren. However, clearly we must recognize that IDPs have a qualitatively lower degree of structural order than folded proteins and they are more competent to perform their functions through frequent disorder-to-order transitions [22,23,24,25,26,27,28].

**Figure 1:**
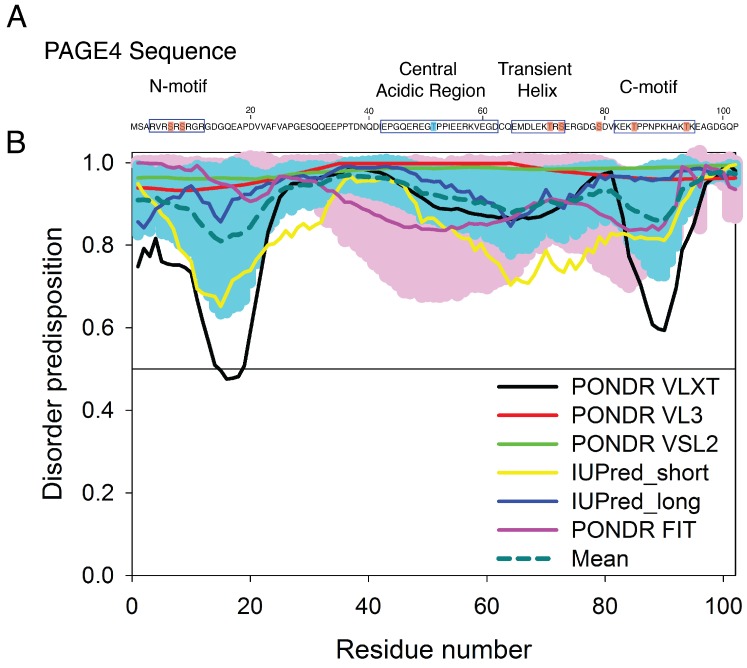
The sequence of prostate-associated gene 4 (PAGE4) and the evaluation of its intrinsic disorder propensity. (**A**) The sequence of PAGE4. The sites phosphorylated by HIPK1 are highlighted in blue, and those phosphorylated sites by CLK2 are highlighted in red. All phosphorylated sites were modeled in the AAWSEM simulation [35]. The N-motif (residues 4–12), Central acidic region (residues 43–62), transiently helical region (residues 65–73) and C-motif (residues 82–95) are also indicated by square box. (**B**) The disorder propensity of PAGE4 was calculated by several per-residue disorder predictors, such as PONDR^®^ VLXT (black line), PONDR^®^ VL3 (red line), PONDR^®^ VSL2 (green line), IUPred_short (yellow line), IUPred_long (blue line) and PONDR^®^ FIT (pink line). The dark cyan dashed line shows the mean disorder propensity calculated by averaging disorder profiles of individual predictors. Light pink and cyan shadows around the PONDR^®^ FIT and mean curve show the error distribution. In these analyses, the predicted intrinsic disorder scores above 0.5 were considered to correspond to the disordered regions.

Prostate-associated gene 4 (PAGE4) is an archetypal IDP implicated in human prostate cancer (PCa) [29,30] (Figure 1). PAGE4 binds to and potentiates the oncoprotein c-Jun, which heterodimerizes with members of the Fos family to form the Activator Protein-1 (AP-1) transcription factor complex [31,32]. AP-1 is a negative regulator of the androgen receptor (AR) [33]. In PCa cells, the interactions among PAGE4, AP-1, Fos, and AR comprise a regulatory circuit module. Theoretical studies suggest that the nonlinear dynamics of this circuit underlie phenotypic switching in PCa cells [34,35]. Bioinformatic algorithms predict that the PAGE4 protein is expected to be highly disordered [36]. Figure 1 represents results of the multiparametric analysis of human PAGE4 intrinsic disorder predisposition by four algorithms from the PONDR family, PONDR^®^ FIT, PONDR^®^ VLXT, PONDR^®^ VSL2, and PONDR^®^ VL3 [37,38,39,40,41], as well as the IUPred web server for prediction of short and long disordered regions [42]. Furthermore, the outputs of all these predictors were averaged to generate the mean disorder profile, since averaging usually increases the predictive performance compared to using any single predictor [43,44]. According to these analyses, PAGE4, although highly disordered, is expected to possess several regions with somewhat increased propensity to order (most notably residues 13–19 and 86–92, and to a lesser degree 49–61). Nuclear magnetic resonance (NMR) experiments also indicate that PAGE4 has metastable secondary structures (Figure 9 of [45]). Two kinases are found to modulate the structure of the PAGE4 ensemble by phosphorylation, specifically, the Homeodomain-interacting Protein Kinase 1 (HIPK1) and the CDC-Like Kinase 2 (CLK2) [34]. The phosphorylated PAGE4 ensembles have different conformational preferences; the structures are relatively more compact for the wildtype form (WT-PAGE4) and for the HIPK1-phosphorylated form (HIPK1-PAGE4) but are more expanded in the CLK2-phosphorylated form (CLK2-PAGE4). PAGE4, although plastic compared to folded proteins, is clearly thus not structurally random. In order for the phosphorylation-induced conformational shift of the PAGE4 ensemble to modulate the binding affinity towards its transcriptional partner, the AP-1 complex, there must be some significant degree of order in the structures and motions of PAGE4 to begin with.

The flexibility of IDPs complicates the detailed characterization of their residual structure and their dynamics with currently available experimental methods. On the other hand, several biophysical techniques such as small angle X-ray scattering (SAXS), single-molecule fluorescence resonance energy transfer (smFRET), and nuclear magnetic resonance (NMR) are able to capture aspects of both the instantaneous and the ensemble-averaged properties of IDPs [46,47,48,49]. Specifically, solution X-ray scattering (SAXS) data enable us to determine the ensemble averaged radii of gyration (Rgs) of IDPs. The chemical shifts from NMR experiments give more local information indicating the preferences of parts of IDPs for different secondary structure elements. NMR paramagnetic relaxation enhancement (PRE) experiments can provide important information about long-range contacts. Changes in the energy transfer efficiency in the smFRET measurements encode the changes of distance between the donor and acceptor probes attached at different locations of the proteins, and can thus be utilized to measure the time-resolved dynamics of different domains within IDPs. The information attained from these experiments about the ensembles of structures of IDPs, and their changes upon post-translational modification, is however somewhat limited and insufficient for correlating the ensemble characteristics with changes in function. One can observe the effects on the ensemble arising from a structural change, but one cannot follow the conformational transition itself in most cases. Therefore, to understand these functionally important structural changes, it is useful to combine experimental measurements with structurally detailed computational simulations.

Molecular dynamics (MD) simulations complement experimental studies and can capture detailed structural dynamics of IDPs. Conventional all-atom simulations, in principle, enable the simulation of IDPs in different environmental conditions [50], but the structural plasticity of IDPs requires that a more substantial part of conformational space be covered by simulations than is needed for well-folded proteins. Sampling IDP ensembles presents a demanding challenge for all-atom simulations. Coarse-grained techniques overcome this problem by integrating out some non-backbone degrees of freedom, leading to much higher computational efficiency [51,52,53]. Furthermore, coarse-grained models allow for flexibility in incorporating the effects of post-translational modifications, such as phosphorylation [54,55].

When examining the structural ensembles for IDPs in simulation, one would like to be able to reproduce experimental observables, sparse as they are. Even though these observables are by themselves insufficient for full specification of the structural ensembles of IDPs, they are important benchmarks for reconciling the results of computational modeling with observations. We must bear in mind, however, that the existence of large conformational fluctuations makes it intrinsically difficult to predict certain properties of IDPs [53]. Small changes in modeling parameters are amplified due to their high effective susceptibility to perturbation, an effect that is less pronounced in the case of the less fluctuating class of folded proteins. Moreover, the majority of the force fields used in protein MD simulations have been tuned to minimize deviations from the folded crystal structure targets. Therefore, we might expect them to over-stabilize intra-chain hydrophobic and hydrogen bonding interactions relative to the interactions with the solvent to keep the molecule folded. These issues are reflected in the over-collapsed nature of the ensemble for IDPs that have been obtained in all-atom MD simulations when compared to measurements from SAXS experiments [56]. To correct this problem and to further control these fluctuations, we can tune the modeling parameters [35,56] or add an external compensatory biasing potential based on the experimental measurements to better represent the ensemble in the laboratory [53,57].

## 2. AAWSEM: A Coarse-Grained Modeling Framework to Simulate Intrinsically Disordered Proteins

The associative memory, water-mediated, structure and energy model (AWSEM) is a coarse-grained model whose parameters have been optimized using the principle of minimal frustration as a machine learning algorithm [58,59]. AWSEM has been successfully applied to the prediction of protein folding and protein structures [60,61,62,63,64,65,66], mechanisms of protein aggregation [67,68], as well as protein–protein and protein–DNA interactions [69,70,71]. AWSEM’s Hamiltonian contains energy terms describing bonded and non-bonded interactions, and an associative memory term [60]:

(1) $$V_{\mathrm{total}}=V_{\mathrm{backbone}}+V_{\mathrm{non}-\mathrm{backbone}}+V_{\mathrm{FM}}+V_{\mathrm{elec}}$$

The non-bonded interactions of AWSEM, $V_{\mathrm{non}-\mathrm{backbone}}$, were learned using the principle of minimal frustration, which provides a quantitative framework for ensuring that the interactions between residue pairs that are close in the native state are dominant in the process of protein folding [72,73]. The associative memory term, $V_{\mathrm{FM}}$, motivated by neural network theory [74], modulates the local structures of protein by using as biases input memories that are structures from the protein database or that have been found using the clustered structures from explicitly simulated protein trajectories through atomistic AWSEM (AAWSEM) [63,64]. Since the structures from the protein database are dominated by globular folded proteins, we use atomistic simulations to generate associative memories for the simulations of IDPs using the AAWSEM model.

The use of the coarse-grained AAWSEM, including the explicit treatment of electrostatic interactions, allows us to investigate the dynamics of IDPs where the effect of charges is predominant. Thus, we used AAWSEM to simulate the WT- and two phospho-forms of PAGE4. The phosphorylated residues are explicitly patched with phosphoryl groups in all-atom simulations for the generation of associative memories, and as far as their long-range interactions are concerned are simulated as hyper-charged (−2.0e) glutamic acid (Glu) residues in the subsequent coarse-grained simulations [54,55]. The electrostatic interactions were simulated by using the Debye-H$\ddot{u}$ckel potential [75] in $V_{\mathrm{elec}}$. Details of the simulation setup can be found in [35].

Similar to the case of explicit-solvent force fields, AWSEM simulation with standard parameters produce an over-collapsed structural ensemble of IDPs [53]. The AAWSEM can partially remedy this issue by simulating segments of PAGE4 in an atomistic force field CHARMM36m, which is known to work well for IDPs [56]. The clustered structures from the all-atom simulations were used as associative memories to guide the local structure formation of PAGE4 in the coarse-grained AWSEM simulation. Nevertheless, such a modified all-atom force field can still fail for larger proteins [76], probably due to the failure of the current functional form of the force field to capture the short-range London dispersion interactions of water molecules [77]. Therefore, to control the degree of collapse of the PAGE4 system, we shifted the γ parameters, which define the residue type based non-bonded interactions, in the non-bonded $V_{\mathrm{non}-\mathrm{backbone}}$ term of the AWSEM potential [35]. The modified parameters ensure that the average radius of gyration $R_{g}$ values for WT-PAGE4 from our simulations match those from the SAXS measurements [34]. The AAWSEM model with these modified parameters then quantitatively predicts the average radii of gyration values for the two phosphoforms of PAGE4. This parameter tuning approach shares some similarity with the one in [77] where the dispersion forces of water molecules were tuned by effectively increasing the C6 term of LJ potential. An alternative approach would be to directly add an explicit biasing potential controlling the size of the simulated systems [53]. We stress that, even without explicitly controlling collapse in this way, the model still reproduces qualitatively the major experimental findings, such as the observed expansion of the size of the CLK2-PAGE4 ensemble upon hyper-phosphorylation [35].

## 3. AAWSEM-Based Simulations of PAGE4 Reveal Two Different Kinds of Order Underneath Its Disordered Cloak

### 3.1. Simulation Reproduces the Expansion of PAGE4 upon Hyper-Phosphorylation

**Figure 2:**
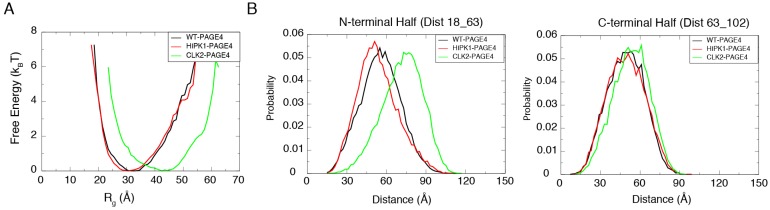
The simulations reproduce the size preference of PAGE4 ensembles at different phosphorylation states. (**A**) The free energy profiles as a function of the radius of gyration ($R_{g}$) of the simulated ensemble of PAGE4. The free energy *F* was calculated as $F=-k_{B}Tlog\left(P\right)$ where *P* is the probability for the protein to have a specific value of the $R_{g}$. The CLK2-PAGE4 exhibits a significant size expansion compared with the HIPK1-PAGE4 and WT-PAGE4. (**B**) The probability distributions for the distances within the two residue pairs that were previously measured in the smFRET experiments [34]. Residues 18 and 63 are located in the N-terminal half while Residues 63 and 102 are in the C-terminal half of PAGE4. The data indicate a more dramatic size expansion in the N-terminal half of CLK2-PAGE4 compared with that in the C-terminal half. Reproduced from [35] with permission.

The free energy profiles generated from the AAWSEM simulations show a shift in the degree of collapse of PAGE4 upon phosphorylation (Figure 2A). HIPK1-PAGE4 (with an average $R_{g}$ = 32.1 Å) has a similar size compared to WT-PAGE4 (with an average $R_{g}$ = 32.9 Å), while CLK2-PAGE4 (with an average $R_{g}$ = 41.8 Å) is greatly expanded in size after hyper-phosphorylation. These combined observations are in line with the experimental SAXS data (WT-PAGE4: 36.2 Å, HIPK1-PAGE4: 34.7 Å and CLK2-PAGE4: 49.8 Å) [34]. In addition, the simulation quantitatively reproduces the smFRET results that the size expansion of CLK2-PAGE4 arises from its expanded N-terminal portion [34] (Figure 2B). The ability to recapitulate experimental results allows one to reliably query the structural details of the WT- and two phospho-forms of PAGE4.

### 3.2. Simulations Reveal Structural Order in PAGE4

**Figure 3:**
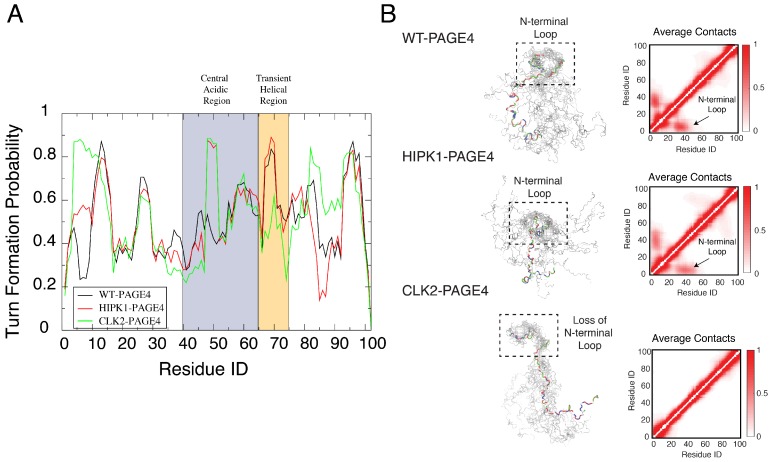
Orderly features behind the disordered PAGE4 ensembles. (**A**) The probability for each residue of PAGE4 to adopt a turn-like structure upon different levels of phosphorylation. The central acidic region and transient helical region are shaded in blue and orange, respectively. The secondary structure was calculated using the Stride algorithm based on the simulated trajectories [78]. Phosphorylations stabilize the turn-like structure in the central acidic region of PAGE4, while hyper-phosphorylation decreases the degree of order in the transiently helical region. (**B**) (**Left**) Representative structural snapshots collected from our simulations generated by AAWSEM. Randomly picked structures are aligned to minimize the root-mean-square deviations (RMSDs) among their N-motifs [79]. (**B**) (**Right**) The average contact maps generated from the simulated ensembles. Contacts are defined as two residues in close spatial proximity to each other. The color bar shows the probability of contact formation. There are non-zero probabilities of contacts formed between the N-motif and the central acidic region in WT-PAGE4 and HIPK1-PAGE4 (indicated by arrows in plots), indicating a metastable structural loop formation in this region. Hyper-phosphorylation eradicates this loop formation in the CLK2 form. Reproduced from [35] with permission.

The simulations uncovered a hidden layer of order underlying the apparent disordered features of PAGE4. Our simulations indicated a change in the preference for forming turn-like structures (as determined using the Stride algorithm [78]) in the central acidic region of PAGE4 upon different levels of phosphorylation (Figure 3A). This structural change is in line with the observations from the NMR experiments (Figure 5 of [45]). Although the AAWSEM simulations agree with the bioinformatic predictions that the overall structure of PAGE4 is disordered, they suggest that some regions of PAGE4 exhibit more structural ordering than do others. Such ordering features are reflected by the population of secondary turn-like structures during simulations. The preference for forming the turn-like structure changes with differing levels of phosphorylation, and the change of the preference of secondary-structure formation suggests an order-to-disorder transition prevalent in the conformational transition of both globular proteins and IDPs [2,17]. Specifically, the increased presence of turn-like structures in the central acidic region of PAGE4 (residues 43 to 62) is consistent with the NMR experiments, which showed a decreased flexibility in the same region upon the phosphorylation of Thr-51 (Figure 9 of [45]). The change in the element of stable turn-like structure also correlates with the change of stability of the binding interface between PAGE4 and its transcriptional partner AP-1 complex. It has been hypothesized that the “transiently helical region” (residues 65–73), a nine-residue region of PAGE4, comprises the binding site towards the AP-1 complex. The simulations of CLK2-PAGE4 suggest a lesser extent of ordering in this region, reflected by a reduced propensity for forming turns after being hyper-phosphorylated (Figure 3A). Therefore, our simulations suggest that there is a hyper-phosphorylation-induced disorder that is associated with the loss of binding affinity of PAGE4 towards its binding partner AP-1 complex. Admittedly, the simulations suggest some changes of the turn-like preference in the N and C termini of PAGE4 upon phosphorylation that were not observed in the NMR experiments. The difference between the simulations and experiment may be attributed to statistical noise coming from poor sampling during the all-atom simulations that are used as input to the AAWSEM simulations.

We also observed the formation of a stable N-terminal loop in WT-PAGE4 and HIPK1-PAGE4, while it disappears in CLK2-PAGE4. The 3D structural ensemble, as well as the constructed average contact map from our simulations (Figure 3B), both show a preference for the N-terminal motif (N-motif, residues 4–12) to form a contact with the central acidic region (residues 43–62) of the WT-PAGE4 and HIPK1-PAGE4 molecules, consistent with the NMR and smFRET experiments (Figure 7 of [45] and Figure 6 of [34]). This stable N-terminal loop formation explains the reduced overall size of PAGE4 in WT-PAGE4 and HIPK1-PAGE4. This loop formation accounts for another level of structural order underlying the disordered dynamics of this IDP molecule.

### 3.3. Collective Motions of PAGE4 Are Associated with Its Functions

**Figure 4:**
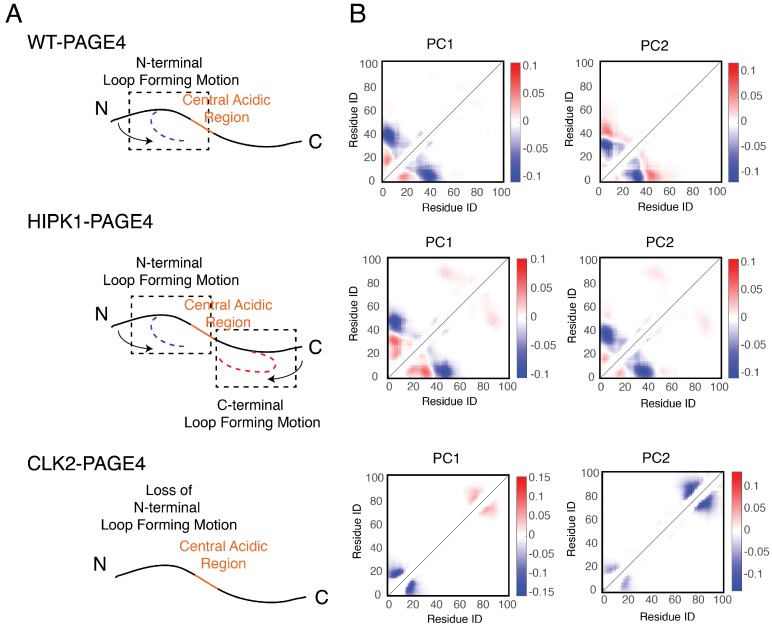
The collective motions revealed from the principal component analysis of PAGE4 simulations are shown. (**A**) Representative cartoon summarizes the collective motions of different phospho-forms of PAGE4. (**Top**) WT-PAGE4 has a collective motion of contacts formed between the N-terminal end and the central acidic region, resulting in a regulated loop formation. (**Middle**) In addition to that, HIPK1-PAGE4 has another loop motion in the C-terminal end that is anti-correlated with that in the N-terminus. (**Bottom**) Hyper-phosphorylation causes the loss of N-terminal loop motion in CLK2-PAGE4. (**B**) The top two principal component modes generated by the contact-based principal component analysis. We plot the coefficients of the first two principal components PC1 and PC2. Larger coefficients indicate a more significant variation of contact formation in that specific principal mode. The relative sign (shown in colors) of two coefficients corresponds to either correlated (same sign) or anti-correlated (opposite signs) formation of contacts. Here, in HIPK1-PAGE4, the C-terminal loop formation has an anti-correlated behavior compared with the N-terminal loop formation. When PAGE4 becomes hyper-phosphorylated, CLK2-PAGE4 loses both N- and C-terminal motion in the first two principal modes. Reproduced from [35] with permission.

In addition to the residual structural order described above, organized dynamics of PAGE4 is revealed by principal component analysis (PCA) of the AAWSEM simulations. We performed PCA based on the contact maps of the simulated ensembles [54,55]. This analysis allows one to classify the collective motions and to understand how correlated movements of different domains of PAGE4 allow long-range interactions to form. The analysis (Figure 4) shows that there are correlated motions at the N-terminal half of WT-PAGE4, where the positively charged N-motif forms a loop with the central acidic region of the protein (shown as blue blobs of the first two principal modes in the top panel of Figure 4B). Interestingly, when WT-PAGE4 becomes phosphorylated by HIPK1, the molecule acquires a second type of motion involving loop formation in the C-terminus (C-motif, residues 82 to 95). The C-terminal motion is anti-correlated with the movement of the N-terminus (shown as additional red blobs of the first two principal modes in the middle panel of Figure 4B). This anti-correlation suggests that the two termini take turns forming a loop with the central acidic region of HIPK1-PAGE4. After PAGE4 is hyper-phosphorylated into CLK2-PAGE4, however, the overall magnitude of disorder increases accompanied by a loss of both types of correlated motions, except for the correlated local motions among these residues that are close in sequence, reflected by randomization of the long-range PC pattern (bottom panel of Figure 4B). The motions associated with the formation of loops by the N- and C-termini of the WT- and HIPK1-PAGE4 may facilitate the binding of PAGE4 to its cognate DNAs or the AP-1 protein complex. The structural plasticity of N- and C-termini enlarges the scope of interactions for PAGE4 to find its binding partners, while looping motion assists in the following binding processes. Such a mechanism is reminiscent of the “fly-casting” motion frequently observed in the studies of IDPs, where the plasticity of the proteins allows them to enlarge their scope of interactions and lowers the free energy barriers for IDPs for finding their binding partners [80,81]. After hyper-phosphorylation of PAGE4, however, a more randomized CLK2-PAGE4 loses its ability to approach and bind to its transactivation partners, resulting in a the loss of function and the degradation of PAGE4 in the end.

## 4. From Structure to Function: PAGE4 Conformational Switching May Underlie Therapy Resistance in Prostate Cancer (PCa)

### 4.1. PAGE4 Conformational Switching Can Give Rise to Oscillations between an Androgen-Dependent Cell Phenotype and an Androgen-Independent Cell Phenotype

The conformational switching of PAGE4 can have important consequences in regulating cellular plasticity in the context of PCa. Double-phosphorylated PAGE4 (HIPK1-PAGE4) can potentiate c-Jun, hence leading to suppression of the androgen receptor (AR) activity. Cells resistant to androgen-deprivation therapy (ADT) typically have high levels of androgen receptor activity [34]. Therefore, high levels of HIPK1-PAGE4 typically correspond to an androgen-dependent (AD) cell phenotype that is sensitive to standard treatment for PCa such as ADT. Conversely, hyper-phosphorylated PAGE4 (CLK2-PAGE4) does not modulate AR activity, and high CLK2-PAGE4 levels can lead to an androgen-independent (AI) cell phenotype that is resistant to androgen deprivation [34].

**Figure 5:**
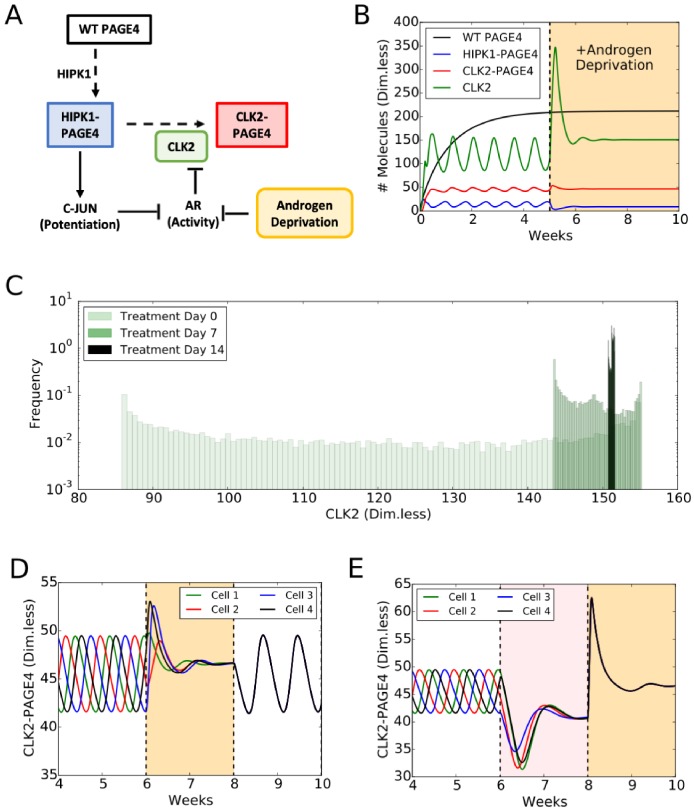
PAGE4 conformational switching gives rise to cell phenotypic oscillations which are suppressed by Androgen Deprivation treatments. (**A**) The PAGE4 phosphorylation circuit and its connection with androgen receptor (AR) activity. Wild-type PAGE4 is double-phosphorylated at two residues by HIPK1 kinase, and HIPK1-PAGE4 is hyper-phosphorylated by the CLK2 kinase. CLK2 is downregulated by AR, which in turn is inhibited by HIPK1-PAGE4 via the intermediates c-Jun. Androgen Deprivation treatment is introduced as an inhibitory signal on AR activity. (**B**) Temporal dynamics of the cellular level of WT PAGE4, HIPK1-PAGE4, CLK2-PAGE4 and CLK2. Without androgen-deprivation therapy (ADT), the oscillatory behavior exhibits a period of approximately one week (left area without shading). ADT (orange-shaded area) quenches oscillations within approximately two weeks. WT PAGE4, HIPK1-PAGE4, CLK2-PAGE4 and CLK2 are represented in dimensionless units. (**C**) Distribution of CLK2 intracellular levels in a simulated cohort of 10,000 prostate cancer (PCa) cells. In the absence of treatment, the distribution of CLK2 levels is broad (“Day 0” case). One week of treatment considerably shrinks the distribution (“Day 7” case). After two weeks of treatment, all cells have a similar level of CLK2 (“Day 14” case). (**D**) Temporal dynamics of CLK2-PAGE4 in four initially unsynchronized cells under intermittent ADT. The orange shading represents the periods of ADT. (**E**) Temporal dynamics of CLK2-PAGE4 in four initially unsynchronized cells under the BAT. The pink and orange shadings represent the periods of AR overexpression and ADT, respectively. Reproduced from [35] with permission.

A mechanism-based mathematical model helps to unravel the role of PAGE4 dynamics in regulating cellular plasticity. The model considers: (1) the different structural conformations of PAGE4: wild-type PAGE4 (WT-PAGE4), HIPK1-PAGE4, and CLK2-PAGE4; and (2) their modulation of AR activity. In the model, the HIPK1 kinase catalyses the switch from WT-PAGE4 to HIPK1-PAGE4, while the CLK2 kinase catalyses the conversion of HIPK1-PAGE4 into CLK2-PAGE4 (Figure 5A). Furthermore, HIPK1-PAGE4 indirectly inhibits AR activity via c-Jun as already discussed. AR activity, however, suppresses the expression of the CLK2 kinase, hence giving rise to a negative feedback between HIPK1-PAGE4 and CLK2 (Figure 5A). This negative feedback can result in oscillations between an AD cell phenotype with (high HIPK1-PAGE4, low CLK2-PAGE4) and an AI cell phenotype with (low HIPK1-PAGE4, high CLK2-PAGE4) (Figure 5B, non-shaded region). These oscillations are largely robust to parameter variation; details of the model setup can be found in [35]. The half-life of WT-PAGE4 is approximately 150 hours, which represents the longer reaction timescale in the system [32]. This timescale is reflected in an oscillation period of approximately one week (Figure 5B). Most androgen-deprivation therapies are applied on a similar timescale of one to several weeks [82,83], therefore indicating the potential role of interactions between treatments and the PAGE4 signaling axis. It should be noted that this prediction about repeated transitions between different cell states needs to be experimentally validated. Further, some modifications in the model topology introduced by additional players can alter the system dynamics so as to be multistable [84], which can also enable cell-state transitions in the presence of biological noise [85].

### 4.2. Androgen Deprivation Restricts the Phenotypic Heterogeneity by Damping PAGE4 Oscillations

The framework has been extended to include a constant inhibitory signal on AR activity (Figure 5A) in order to model the application of Androgen Deprivation Therapy (ADT), the standard care of treatment for locally advanced and metastatic PCa [82]. One finds that ADT quenches the oscillatory dynamics and can stabilize the AI, therapy-resistant cell phenotype (Figure 5B, orange-shaded part). To understand the implications of ADT at a cell population level, PAGE4 oscillations in a cohort of 10,000 PCa cells were simulated. In the cell population, oscillations are not synchronized: at any given time, some cells will be in the AI phase of the PAGE4 oscillation cycle, while others will be in the AD phase of the PAGE4 oscillation cycle. Therefore, the distribution of intracellular CLK2 (or any other variable in the model) in the population is quite broad (Figure 5C, Day 0 case). Introducing ADT, however, restricts cell heterogeneity by forcing the cells generally to have a very similar intracellular CLK2 level in approximately two weeks (Figure 5C, Day 7 and Day 14 cases). Thus, the model predicts that ADT can reduce the extent of non-genetic heterogeneity of a PCa cell population. Importantly, this model does not explicitly consider cell death. It is reasonable to hypothesize that, in the presence of ADT, some PCa cells would undergo therapy-induced apoptosis, but that any surviving cells can exhibit resistance. Therefore, the model predicts that the surviving PCa cell population is likely to consist of a much more homogeneous cohort of androgen-independent (or ADT-resistant) cells, due to synchronization of oscillations across the population.

A number of promising treatments for PCa besides ADT have been proposed in recent years, including intermittent ADT [82]. Intermittent ADT considers periodic ADT separated by “drug holiday” periods when no treatment is applied. As already discussed, a timescale of a week of ADT is sufficient to suppress oscillations in a cell population. These oscillations, however, emerge again during the holiday periods when AR activity is not inhibited (Figure 5D). Intriguingly, the on-off treatment cycle synchronizes the oscillations in cells that were initially unsynchronized (Figure 5D). This prediction, which still needs detailed experimental validation, lends further support to the idea that treatments such as ADT can restrict the phenotypic heterogeneity of a PCa cell population. Several independent investigations have considered various durations of ADT and holiday periods in both xenograft models and the clinic [86]. Based on our simulation results, a period of at least one week appears necessary since the switch of cell states is on a similar timescale.

As another example of alternative therapy, Bipolar Androgen Treatment (BAT), alternates two weeks of AR overexpression and two weeks of regular ADT [82]. Similar to the intermittent ADT, BAT is predicted to synchronize PCa cells that are otherwise out of phase prior to treatment (Figure 5E). Interestingly, the period of AR overexpression enforces an androgen-dependent phenotype (pink-shaded area in Figure 5E), while the two weeks of ADT lead to the already observed AI phenotype (orange-shaded area in Figure 5E). Therefore, the effectiveness of this treatment might lie in its ability to establish a treatment-sensitive phenotype first, which afterwards makes PCa cells more vulnerable to ADT.

## 5. Discussion and Conclusions

Intrinsically disordered proteins (IDPs) play a number of key roles in mediating interactions among the molecular components of the cell [2]. Their malleable structural properties enlarge their scope of interactions and lower the thermodynamic barriers for finding and associating with their interacting partners [80], which ranges from proteins to DNA. Intrinsically disordered proteins stand on the brink of stability and thus have more flexibility in sampling relevant conformational space [18]. On the other hand, an IDP is certainly not a completely disordered Gaussian polymer chain. Its underlying sequences encodes a certain level of order manifested in its specificity for the purpose of its function and stabilization upon binding to its interaction partners [7]. Even for a protein such as PAGE4, which has been identified as a near-complete random coil by bioinformatic tools [36], there are underlying structural features that confer on it the ability to respond differently to different levels of post-translational phosphorylation [35]. Therefore, under the cloak of its generally disordered nature, it is the residual order that bestows each IDP with its characteristic functions in the cell.

The presence of order-to-disorder conformational transitions with functional implications is not a unique trait of IDPs. Instead, we can gain insights from multiple examples well-studied in the realm of globular proteins as well [87,88,89,90,91]. On a local scale, a “cracking” motion features an order–disorder–order transition that is utilized by some proteins as a way to lower the barrier of conformational transition, or to facilitate the allosteric transitions [87,88,92]. On a global scale, a complete structural rearrangement of the system is sometimes needed to deliver the functional parts of a protein into its target location [91]. The plasticity of protein structures typically facilitates such transitions, but it is the ordering of the structure that eventually enables the functions. By the same token, although the sensitivity of IDP structure to perturbations indicates that modeling errors are amplified, the currently available force fields developed for folded proteins may already be accurate enough to capture the essential properties of IDPs, especially their thermodynamics, which is less sensitive. The fact that we can use a slightly modified version of the AWSEM model, which was initially optimized for folding globular proteins [60], to accurately capture the existing experimental data on PAGE4 [35], demonstrates that the key components built into this globular protein-derived force field do not differ too much from the actual forces at work in IDPs. Future work, however, is required to unify the modeling of IDPs and globular proteins for the simulations of an extensive network of protein complexes involving different types of proteins and other critical cellular components.

Another research area that needs further investigation is integrating structural insights into mechanism-based systems biology models. Here, the findings from our systems biology based model for PAGE4 and its interacting partners emphasize the role that conformational dynamics of proteins can play in rewiring regulatory networks, which in turn has important consequences for phenotypic plasticity. Such plasticity is thought to be related to transcriptional noise (i.e., stochastic gene expression [93,94,95]), but our analysis suggests that “conformational noise” may also modulate cell-fate [96]. Particularly, in the context of cancer progression, many oncogenes, tumor-suppressor genes, and regulators of metastatic spread have been identified as IDPs [97,98,99]. Comparing the conformational ensembles of cancer-specific mutant forms with those of their wild-type counterparts can yield important insights for therapeutic strategies.
